# Supplementary material for: Identification of photoperiod-induced specific miRNAs in the adrenal glands of Sunite sheep (Ovis aries)
Source: Front Vet Sci. 2022 Jul 22;9:888207. doi: 10.3389/fvets.2022.888207 (PMC9354845; doi:10.3389/fvets.2022.888207)
Supplement: Supplementary file 1 [file Data_Sheet_1.ZIP › Supplementary file/miRDeep2 file.zip!/miRDeep2 file/Know_Structure/SPLP42A2/oar-mir-1193.pdf]

miRBase precursor : oar-mir-1193
Total read count : 5
oar-miR-1193-5p read count : 4
oar-miR-1193-3p read count : 0
remaining reads : 1

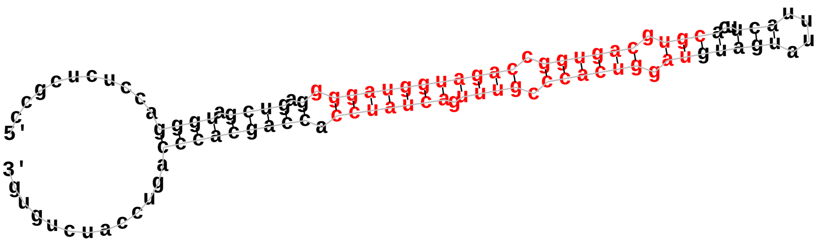

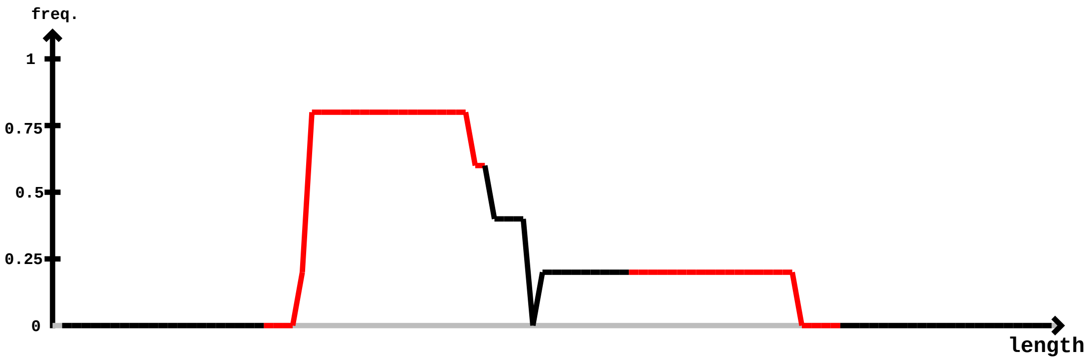


```
                     oar-miR-1193-5p
                                              oar-miR-1193-3p
5'- ccgcucuccaggguagcugaggggaugguagaccggugacgugcacuucauuuaugauguaggucacccguuugacuauccaccagcacccaguccaucugug   -3'    exp
    ..........((((.((((.(.(((((((((((.((((((.((((..(((....))))))).)))))).)))).))))))).)))))))))............    reads      mm        sample
    ........................augguagaccggugacgu.............................................................    1          0         seq
    .........................ugguagaccggugacgugc...........................................................    1          0         seq
    .........................ugguagaccggugacgugcacuu.......................................................    2          0         seq
    .................................................auuuaugauguaggucacccguuugac...........................    1          0         seq
```
